# Evolution of major histocompatibility complex class I genes in the sable *Martes zibellina* (Carnivora, Mustelidae)

**DOI:** 10.1002/ece3.6140

**Published:** 2020-03-11

**Authors:** Baojun Zhao, Xue Zhang, Bo Li, Pengfei Du, Lupeng Shi, Yuehuan Dong, Xiaodong Gao, Weilai Sha, Honghai Zhang

**Affiliations:** ^1^ College of Life Science Qufu Normal University Qufu China; ^2^ College of Wildlife and Protected Area Northeast Forestry University Harbin China

**Keywords:** balancing selection, Carnivora, major histocompatibility complex, *Martes zibellina*, recombination

## Abstract

The molecules encoded by major histocompatibility complex (MHC) genes play an essential role in the adaptive immune response among vertebrates. We investigated the molecular evolution of MHC class I genes in the sable *Martes zibellina*. We isolated 26 MHC class I sequences, including 12 putatively functional sequences and 14 pseudogene sequences, from 24 individuals from two geographic areas of northeast China. The number of putatively functional sequences found in a single individual ranged from one to five, which might be at least 1–3 loci. We found that both balancing selection and recombination contribute to evolution of MHC class I genes in *M. zibellina*. In addition, we identified a candidate nonclassical MHC class I lineage in Carnivora, which may have preceded the divergence (about 52*–*57 Mya) of Caniformia and Feliformia. This may contribute to further understanding of the origin and evolution of nonclassical MHC class I genes. Our study provides important immune information of MHC for *M. zibellina,* as well as other carnivores.

## Graphical abstract

We investigated the molecular evolution of major histocompatibility complex (MHC) class I genes in the sable *Martes zibellina*. We found that both balancing selection and recombination contribute to evolution of MHC class I genes in *M. zibellina*. In addition, we identified a candidate nonclassical MHC class I lineage in Carnivora, which may have preceded the divergence (about 52–57 mya) of Caniformia and Feliformia.

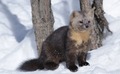

## 1 INTRODUCTION

The major histocompatibility complex (MHC) plays a crucial role in the adaptive immune system (Klein, 1986). There are two major types of MHC gene families, class I and class II, which encode cell surface glycoproteins that regulate the immune response. MHC class II molecules are heterodimers formed by an α chain and a β chain, which both contribute to presenting peptides derived from extracellular proteins to the CD4^+^ T‐helper cells (Castellino, Zhong, & Germain, 1997). The α1 domain and β1 domain are the regions containing the antigen‐binding sites (ABSs) in α chain and β chain, respectively. MHC class I molecules are heterodimers consisting of an α chain and a non‐MHC molecule, β2 microglobulin. The α chain contains a cytoplasmic tail, a transmembrane domain, and three extracellular domains designated α1, α2, and α3 (Bjorkman & Parham, 1990) that are encoded by exons 2, 3, and 4. The α1 domain and α2 domain are the regions containing ABSs in α chain of MHC class I molecule. MHC class I genes are further classified into classical and nonclassical MHC class I genes. The classical MHC class I molecules are encoded in all somatic cells and are responsible primarily for triggering adaptive immune response by presenting endogenously derived peptides to CD8^+^ cytotoxic T cells (Neefjes, Jongsma, Paul, & Bakke, 2011). In contrast to classical MHC class I genes, nonclassical MHC genes have tissue‐specific expression, low levels of polymorphism, and lower expression levels (Braud, Allan, & McMichael, 1999; Rodgers & Cook, 2005; Stroynowski & Lindahl, 1994).

Major histocompatibility complex genes are considered to be the most polymorphic in the vertebrate nuclear genome (Horton et al., 2004). And the localization of polymorphism is largely within the region encoding the ABSs of the MHC class I and class II molecules (Yeager & Hughes, 1999). The mechanisms that produce plentiful MHC variation primarily involve balancing selection, for instance, overdominance, and negative frequency‐dependent selection. The overdominance hypothesis, which was proposed by Doherty and Zinkernagel (1975), suggests that heterozygosity at MHC loci could significantly enhance immune competence, as heterozygotes could recognize a wider range of antigens derived from multiple pathogens and therefore have a higher relative fitness than either homozygote (Piertney & Oliver, 2006). The negative frequency‐dependent selection holds that rare alleles have a selective advantage over common host alleles because pathogens tend to adapt the common alleles (Schad, Ganzhorn, & Sommer, 2005).

The MHC region is also thought to evolve under birth‐and‐death evolution. In the process, novel genes are created by gene duplication and some duplicate genes remain in the genome for a long time, whereas others are completely lost from the genome or become nonfunctional genes (pseudogenes) due to deleterious mutations (Nei, Gu, & Sitnikova, 1997). Compared to MHC class II genes, the rate of birth‐and‐death evolution in MHC class I genes appears faster, and as a consequence, it is difficult to establish orthologous relationships of MHC class I genes among mammalian orders (Abduriyim, Zou, & Zhao, 2019b; Kuduk, Babik, et al., 2012; Takahashi, Rooney, & Nei, 2000). In addition, recombination has been considered as an important mechanism, which contributes to the high divergence of MHC class I genes between closely related species and the diversity of MHC genes (Gaigher et al., 2018; Nei & Rooney, 2005; Zhao et al., 2013).

Mustelidae is the largest and most diverse family of order Carnivora (Hosoda et al., 2000). Members of the Mustelidae show a tremendous range of ecomorphologic diversity, from species that are fossorial to those that are semi‐ or completely aquatic (Wei, Zhang, Wu, & Sha, 2019; Wozencraft, 1993). The sable *Martes zibellina* (Linnaeus, 1758), genus *Marten*, is a medium‐sized carnivore distributed in all taiga zoogeographical zones of Eurasia, primarily in Russia, China, Mongolia, North Korea, Kazakhstan, and Japan (Li et al., 2013; Monakhov, 2011). In China, it occurs in northeast China (Greater Khingan Mountains, Lesser Khingan Mountains, and Changbai Mountains) and Xinjiang Uygur Autonomous Region (Altai Mountains). *Martes zibellina* has historically been hunted for its prized fur. As a consequence of deforestation and commercial hunting, the distribution range and abundance of this species have decreased rapidly in northeast China since the 1950s (Zhang et al., 2017). Large‐scale regional extinctions of *M. zibellina* have aroused the attention of wildlife management department in China, and it was listed as class I national protected species in 1989.

Because of the important role of MHC genes, the study of MHC genes in wild populations could provide useful information regarding immunological adaptation and fitness (Manlik et al., 2019). Studies of MHC class II genes have been reported in many species of Mustelidae (Bowen, Aldridge, Miles, & Stott, 2006; Nishita et al., 2015; Sin, Dugdale, Newman, Macdonald, & Burke, 2012b); however, the studies of MHC class I in Mustelidae are limited to genus *Meles* (Abduriyim, Nishita, et al., 2019; Sin, Dugdale, Newman, Macdonald, & Burke, 2012a). Moreover, MHC has been shown to be associated with mate choice in many species, which can be used to evaluate potential mate (Baratti et al., 2012; Cutrera, Fanjul, & Zenuto, 2012; Sin et al., 2015). Studies of MHC genes may contribute to breeding and conservation of endangered species. In addition, the rapid turnover of genetic loci make the evolution of MHC genes an intriguing subject of study (Piontkivska & Nei, 2003). Previous studies have noted that some nonclassical MHC genes have existed in genome for a long time (Nei et al., 1997). For instance, in primate species, the nonclassical F locus has existed for at least 46*–*66 Mya while the A, B, and C loci have appeared at least 14*–*19, 10*–*15, and 13*–*17 Mya, respectively. Although some nonclassical MHC genes have been identified according to the tissue‐specific expression patterns, abnormal exon, and limited polymorphism in some carnivores (Burnett, DeRose, Wagner, & Storb, 1997; Zhu et al., 2012), there is little research about the orthologous relationships. In this study we (1) isolated the exon 2/α1 domain*–*exon 3/α2 domain (including intron 2) of MHC class I genes from 24 sables in two geographic areas in northeast China; (2) analyzed signals of natural selection and recombination in MHC class I genes of *M. zibellina*; and (3) performed phylogenetic analyses to assess the evolutionary relationship of MHC class I genes in Carnivora. The results of this study may provide important immune information for *M. zibellina* and will contribute to conservation of this species as well as other carnivores.

## 2 MATERIAL AND METHODS

### 2.1 Sample information and DNA extraction

**Figure 1:**
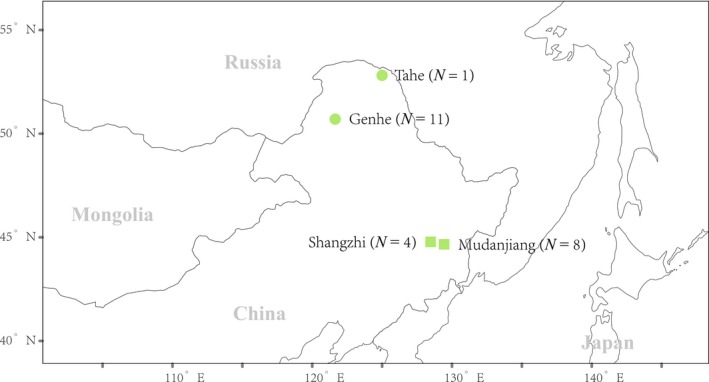
Map of the sampling locations in China. The individuals, which from the areas (Genhe and Tahe) near the Greater Khingan Mountains, were indicated with the circles. The individuals, which from the areas (Shangzhi and Mudanjiang) between the Lesser Khingan Mountains and the Changbai Mountains, were indicated with the squares

We used 24 tissue samples (Skeletal muscle) from sables, of which, 12 were from the areas (Genhe and Tahe) near the Greater Khingan Mountains, others were from the areas (Shangzhi and Mudanjiang) between the Lesser Khingan Mountains and the Changbai Mountains (Figure 1). Total genomic DNA of *M. zibellina* was extracted from tissue samples using the DNeasy Blood & Tissue DNA Extraction Kit (Qiagen) following the protocol of the manufacturer.

### 2.2 PCR amplification

We amplified part of MHC class I gene (exon 2/α1 domain*–*exon 3/α2 domain, including intron 2) from the samples by PCR using two pairs of primers. The first pair of primers were from Meme‐MHCIex2F and Meme‐MHCIex3R (Sin et al., 2012a). The second pair of primers (Mazi‐MHCIex2F: 5′‐GCTCCCACTCCCTGAGGTATTWC‐3′; Mazi‐MHCIex3R: 5′‐GCGCAGCAGCGWCTCCTT‐3′), which recognize highly conserved region of the MHC class I genes, were designed based on alignments consisting of the sequences from the NCBI and the sequences obtained from the first pair of primers (Figure S1). PCR amplifications were performed in 25 μl reaction volumes containing 10× PCR Buffer (Mg^2+^ plus; Takara), 200 µM of dNTP Mixture (Takara), Bovine Serum Albumin (Takara), 50*–*200 ng of total DNA, 0.5 µM of each primer, and 1 U of Takara Taq. Cycling condition in Applied Biosystems (ABI) 9700 Thermal Cycler was 5 min at 94°C; 35 cycles of 30 s at 94°C, 30 s at 60°C, 60 s at 72°C, and a final hold at 4°C. The PCR products were electrophoresed on 1% agarose gel and visualized using ultraviolet light. Target bands (around 750 bp) were excised from the gel and purified using QIAquick Gel Extraction Kit (Qiagen).

### 2.3 Cloning and sequencing

Purified PCR products were ligated into pMD 18‐T Vector (Takara). Recombinant DNA was transformed into *E. coli* DH5α Competent Cells (Takara), which were then plated onto LB plates and grown overnight at 37°C. Blue‐white selection was used to select positive clones. PCRs were performed on positive clones using M13 forward and reverse primers. The positive clones were sequenced by Sangon Biotech (Shanghai) using ABI3730XL DNA Analyzer. Between 35 and 40 clones were sequenced for each individual. Each clone was sequenced once. The nucleotide sequences were manually trimmed and aligned in MEGA 7.0 (Kumar, Stecher, & Tamura, 2016). The sequences obtained by the two pairs of primers were trimmed to the same length. We recognized a positive clone sequence as a MHC class I sequence if the sequence was found in two independent PCR reactions from a single individual or appeared in at least two individuals (Liu et al., 2017; Sin et al., 2012a). All selected sequences were further compared with the identified MHC class I genes using the program of BLAST on NCBI. The sequences of *M. zibellina* which showed any signs of insertion, deletion, or premature stop codons in α1 domain or α2 domain were identified as presumed pseudogene sequence, and others were considered as the presumably functional sequence (Abduriyim, Nishita, et al., 2019). The MHC class I sequences identified in this study were named according to the nomenclature conventions (Klein et al., 1990).

### 2.4 Data analysis

The average pairwise Poisson‐corrected amino acid distances and the nucleotide distances (Kimura 2‐parameter model—K2P) were computed in MEGA7.0 (Kumar et al., 2016). Standard errors were obtained through 1,000 bootstrap replicates. The nucleotide diversity (*π*) was calculated using DnaSP. Nonsynonymous (*d*_N_) and synonymous (*d*_S_) substitution rates were computed with MEGA 7.0 according to the Nei–Gojobori method (Nei & Gojobori, 1986) with the Jukes–Cantor correction. Standard error estimates were obtained through 1,000 bootstrap replicates. These calculations were performed separately for the ABSs and non‐ABSs which were determined by Bjorkman et al. (1987). Z tests were performed in MEGA 7.0. The program CODEML in PAML 4.9 was used to detect positively selected sites (PSS) in α1 domain and α2 domain, which are indicated where the ratio *ω* (*d*_N_/*d*_S_) is larger than 1 (Yang, 2007). Two different models, M7 (beta) and M8 (beta and *ω*), were tested. The likelihood ratio tests (LRT), which compare twice the difference of the log‐likelihood ratios (2Δln*L*) to a *chi‐square* distribution, were used to determine whether the alternative model (M8) provided a significantly better fit than the null model (M7). Bayes empirical Bayes posterior probabilities (>0.95) were used to identify the PSSs in the M8.

Recombination analyses were implemented for the nucleotide alignment spanning α1 domain, intron 2, and α2 domain in Recombination Detection Program version 4 (RDP4). Several methods, including RDP (Martin & Rybicki, 2000), GENECONV (Padidam, Sawyer, & Fauquet, 1999), Chimaera (Posada & Crandall, 2001), MaxChi (Smith, 1992), BootScan (Martin, Posada, Crandall, & Williamson, 2005), SiScan (Gibbs, Armstrong, & Gibbs, 2000), and 3Seq (Boni, Posada, & Feldman, 2007), were used to detect recombination events. In addition, the online GARD tool, provided by the Datamonkey web server (http://www.datamonkey.org/), was used to assess the presence of recombination signals (Kosakovsky Pond, Posada, Gravenor, Woelk, & Frost, 2006). Although gene conversion and recombination are mechanistically different processes, the effects on polymorphism are similar in sequences of limited length (Burri, Hirzel, Salamin, Roulin, & Fumagalli, 2008). We therefore do not distinguish between them and refer to them as recombination in the broad sense (Sin et al., 2012a).

Phylogenetic networks have an important role to play in the reconstruction of evolutionary history (Huson & Bryant, 2006). Compared with phylogenetic trees, phylogenetic networks can effectively evaluate evolutionary relationships involving gene duplication and recombination which are known to affect MHC gene evolution (Abduriyim, Zou, et al., 2019; Miller & Lambert, 2004; Zhao et al., 2013). Phylogenetic analyses were separately carried out on the nucleotide alignment of α1 domain and α2 domain against homologous sequences from other carnivore species available in NCBI. Most of these sequences are from previous studies of MHC class I, including domestic cat (*Felis catus*) (Yuhki, Mullikin, Beck, Stephens, & O'Brien, 2008), wolf (*Canis lupus*) (Liu et al., 2017), domestic dog (*Canis lupus familiaris*), giant panda (*Ailuropoda melanoleuca*) (Zhu et al., 2012; Zhu, Wan, Yu, Ge, & Fang, 2013), harbor seal (*Phoca vitulina vitulina*) (Hammond, Guethlein, Norman, & Parham, 2012), gray seal (*Halichoerus grypus*), tiger (*Panthera tigris tigris*) (Pokorny, Sharma, Goyal, Mishra, & Tiedemann, 2010), ocelot (*Leopardus pardalis*) (Yuhki & O'Brien, 1994), cheetah (*Acinonyx jubatus*), and Eurasian bargers (*Meles meles*, *M*. *canescens*, *M*. *leeucurus*, and *M*. *canescens*) (Abduriyim, Nishita, et al., 2019; Sin et al., 2012a). Other sequences are from the Genome Data. The species covered are mainly from Felidae, Ursidae, Otariidae, Odobenidae, Phocidae, Mustelidae, Hyaenidae, and Canidae. Neighbor‐Net method in SplitsTree 4.14.8 was used to analyze the phylogenetic relationships. Neighbor‐Net networks were constructed according to uncorrected *P*‐distances. 1,000 bootstrap replicates were conducted to estimate the nodal support, and the nodal support values (>75%) were displayed in the phylogenetic networks. To further identify the nonclassical MHC class I lineage in Carnivora, we constructed maximum likelihood (ML) phylogenetic trees using IQ‐TREE (Nguyen, Schmidt, Haeseler, & Minh, 2015). The best models for α1 domain and α2 domain were determined using ModelFinder (Kalyaanamoorthy, Minh, Wong, Haeseler, & Jermiin, 2017). We conducted 1,000 bootstrap replicates to estimate the support. Values greater than 75% were indicated in the ML phylogenetic trees.

## 3 RESULTS

### 3.1 Identification of MHC class I

A total of 900 clones were sequenced from 24 individuals of *M. zibellina.* The number of clones sequenced per individual ranged from 35 to 40, with an average of 37.5 clones. There were 311 clones which were found in two independent PCR reactions from a single individual or appeared in at least two individuals (Table S1). The final aligned MHC class I dataset included α1 domain (246 bp), intron 2 (variable; 192*–*221 bp), and α2 domain (255 bp). We identified 26 distinct MHC class I alleles (Table S2), including 12 presumably functional sequences (Figure S2) and 14 pseudogene sequences (accession numbers: MN274976*–*MN275001). All the sequences showed a high similarity to the MHC class I genes in GenBank. The numbers of presumably functional sequences found in a single individual ranged from one to five, indicating that at least one to three loci exist in *M. zibellina*.

The Mazi‐MHCI*PS01–*PS04* showed a premature stop codon at amino acid position 75 encoded from α1 domain. The nucleotide deletions caused frameshift and premature stop codons were detected in the Mazi‐MHCI*PS06–PS12. The Nucleotide deletions, which caused loss of 4 amino acids, were detected in Mazi‐MHCI*PS13 and *PS14. Both nucleotide insertions and deletions were detected in the α2 domain of Mazi‐MHCI*PS05. We excluded these presumed pseudogene sequences in analyses of selection and recombination. Among 26 MHC class I alleles from *M. zibellina,* there were 13 unique intron 2 sequences, with length variants of 192, 193, 194, 198, 204, 208, 209, and 221 bp (Figure S3). The number of variable sites of α2 domain was higher than that of α1 domain, but the α1 domain had fewer synonymous substitutions (Table 1). The average pairwise nucleotide and amino acid distances were similar between α1 domain and α2 domain (Table 2). However, the average pairwise nucleotide and amino acid distances at ABSs of α1 domain were higher than ABSs of α2 domain. In α1 domain and α2 domain, the average nucleotide diversities of ABSs were higher than the average nucleotide diversities of non‐ABSs.

**Table 1:** Sequence polymorphism of MHC class I genes of *Martes zibellina*

Domain	α1 domain	α2 domain
Variable sites	53	63
Parsim informative sites	47	48
Mutations	61	70
Synonymous	8	22
Nonsynonymous	33	35
Number of amino acids	82	85
Polymorphic amino acid residues	29	30

**Table 2:** The average rates of nonsynonymous (*d*_N_) and synonymous (*d*_S_) substitutions and the result of *Z* test, the average nucleotide diversity (*π*), the average nucleotide distances (*d*_nt_), and amino acid distances (*d*_aa_) for ABSs, non‐ABSs, and all sites in MHC class I α1 domain and α2 domain for *Martes zibellina*

Domain	Sites	*d*_N_	*d*_S_	*Z*	*p*	*ω*	*π*	*d*_nt_	*d*_aa_
α1	All sites	0.105 (0.021)	0.078 (0.025)	1.040	.150	1.356	0.091	0.051 (0.010)	0.191 (0.035)
	ABSs	0.298 (0.076)	0.127 (0.076)	1.848	**.034**	2.345	0.204	0.141 (0.045)	0.572 (0.162)
	Non‐ABSs	0.070 (0.018)	0.068 (0.028)	0.061	.476	1.026	0.065	0.034 (0.009)	0.126 (0.029)
α2	All sites	0.090 (0.018)	0.165 (0.039)	−1.792	1.000	0.546	0.099	0.052 (0.010)	0.170 (0.031)
	ABSs	0.219 (0.088)	0.184 (0.131)	0.300	.383	1.187	0.176	0.057 (0.030)	0.366 (0.131)
	Non‐ABSs	0.065 (0.013)	0.163 (0.042)	−2.265	1.000	0.395	0.082	0.051 (0.011)	0.134 (0.029)

Note

The standard errors, obtained through 1,000 bootstrap replicates, are in parentheses.

Abbreviation: ABS, antigen‐binding site.

Significant results are highlighted in bold.

### 3.2 Selection and recombination

Considering that the evolutionary history of each domain might have been different, we tested each domain separately for evidence of positive selection. The nonsynonymous substitution rate (*d*_N_) was significantly higher than the synonymous substitution rate (*d*_S_) in the ABSs of the α1 domain (Table 2). Although the M8 model detected PSSs in the α1 domain, the positive selection model M8 did not provide a better fit than the neutral evolution model M7 (Table 3). In the α2 domain, M8 model showed a better fit than M7 model. Two sites were recognized as being under positive selection, and both of them were within the ABSs.

**Table 3:** Inference of positively selected sites (PSSs) for sable MHC class I sequences

Domain	Model	ln *L* value	Parameter estimates	PSSs	LRT	TS value	*p*‐Value
α1	M7	−665.75	*P* = 0.01, *q = 0.02*	Not allowed	M7 vs. M8	3.86	>.05
	M8	−663.82	*P*_0_ = 0.91, *P* = 0.01, *q* = 0.01, *P*_1_ = 0.09, ω = 3.33	40D, **45D**, 66I, 67C, 79Q, **81A**			
α2	M7	−733.53	*P* = 0.19, *q = *0.44	Not allowed	M7 vs. M8	6.10	<.05
	M8	−730.49	*P*_0_ = 0.97, *P* = 0.31, *q* = 0.81, *P*_1_ = 0.03, ω = 7.84	155R, 156L			

Note

The log‐likelihood (ln *L*) values and estimated parameters were computed using CODEML in PAML 4.9. PSSs were inferred in model M8 by Bayes empirical Bayes (BEB) with posterior probabilities (PP) > 95%, and codons with PP > 99% are shown in bold. Codons located at antigen‐binding sites are highlighted with underline. TS value indicates test statistics, TS value = twice the difference of the log‐likelihood ratios (2ΔIn *L*). Degree of freedom is two for all LRTs. *p*‐values were determined by comparison of TS to a *chi‐square* distribution.

Two significant recombination events were detected in the MHC class I sequences of *M. zibellina* in RDP4 program (Table S3). Four recombination breakpoints were detected, one of which was located in α1 domain, two of which were situated in intron 2, and one of which was in α3 domain. In addition, GARD identified a recombination signal in *M. zibellina* MHC class I sequences. A recombination breakpoint (148, <0.01) was detected in α1 domain. The recombination breakpoints identified by RDP4 are not consistent with the recombination breakpoint identified by GARD. The recombination breakpoints identified by these two programs are often inconsistent, probably due to the fact that they use different computational methods.

### 3.3 Phylogenetic analyses

**Figure 2:**
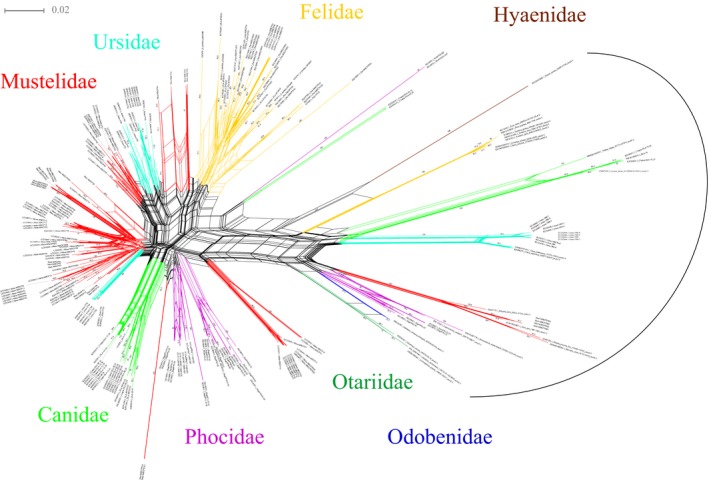
Neighbor‐Net network for MHC class I exon 2/α1 domain sequences from *Martes zibellina* in this study and including other sequences of carnivores obtained from NCBI

**Figure 3:**
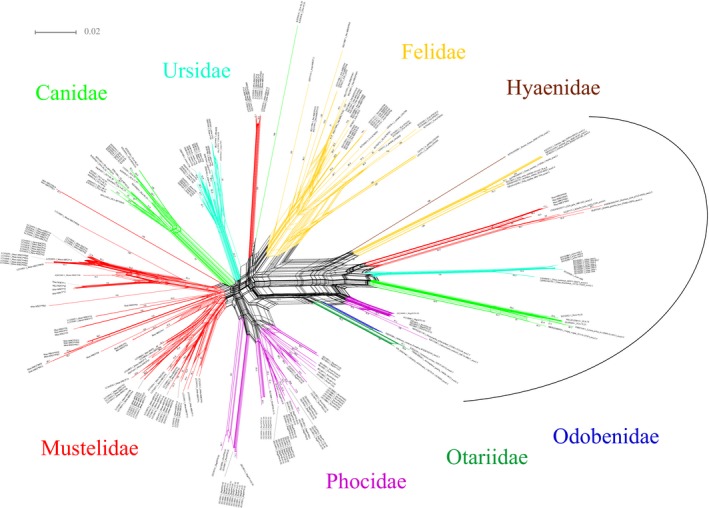
Neighbor‐Net network for MHC class I exon 3/α2 domain sequences from *Martes zibellina* in this study and including other sequences of carnivores obtained from NCBI

The phylogenetic networks of α1 domain and α2 domain showed that most sequences of *M. zibellina* formed a clade with other musteline sequences (Figures 2 and 3). The phylogenetic network of α1 domain was not fully consistent with the phylogenetic tree of α2 domain. For instance, the Mazi‐MHCI*01–04 formed a monophyletic clade in the phylogenetic network of α1 domain. In the phylogenetic networks of α1 domain and α2 domain, the Mazi‐MHCI*PS14 and Mazi‐MHCI*PS14 formed a separate clade. The Mazi‐MHCI*PS01–04 formed a clade with the nonclassical MHC class I genes (*Aime‐1906*, *DLA‐79*) identified in some carnivores (Figures 2 and 3). The ML trees of α1 domain and α2 domain showed that these sequences group into a clade with high bootstrap values (95% for α1 domain; 94% for α2 domain) (Figures S4 and S5).

## 4 DISCUSSION

This is the first study to characterize MHC class I genes in *M. zibellina*. We identified 12 presumably functional sequences and 14 presumed MHC class I pseudogene sequences. The major structural features that distinguish classical MHC class I proteins are present in these presumably functional sequences, such as highly conserved amino acid residues in α1 domain and α2 domain that bind the N‐ and C‐termini of the presented peptide, a threonine (T134) residue for interaction with the TAP (transporter associated with antigen processing) complex (Peace‐Brewer et al., 1996), an N‐linked glycosylation site (N86) in α1 domain, and cysteine (C) residues that form the disulfide bond (Figure S2) (Kaufman, Salomonsen, & Flajnik, 1994; Sin et al., 2012a). These features, together with the extensive polymorphisms we detected in presumably functional sequences (Tables 1 and 2), indicate that most of the sequences from *M. zibellina* are classical MHC class I genes. Mammals usually possess limited number of classical loci (Hammer et al., 2020; Hughes & Nei, 1989). For instance, the human has three (*HLA‐A*, *HLA‐B*, and *HLA‐C*) classical MHC class I genes; Tasmanian devil (*Sarcophilus harrisii*) has three (*Saha‐UA*, *Saha‐UB*, and *Saha‐UC*) classical I genes (Cheng et al., 2012); domestic dog has one (*DLA‐88*) classical MHC class I gene; giant panda has four (*Aime‐C*, *Aime‐F*, *Aime‐I*, and *Aime‐L*) classical MHC class I gene (Pan, Wan, & Fang, 2008; Zhu et al., 2012). In the current study, the number of presumably functional sequences found in a single individual ranged from one to five, which might be at least 1*–*3 loci. This is consistent with other mammalian studies. In addition, we detected a large number of presumed pseudogene sequences in *M. zibellina*. This is in line with the expectation of birth‐and‐death evolution (Nei & Rooney, 2005). One thing to note is that we cannot rule out the possibility that the Mazi‐MHCI*PS13 and *PS14 are nonclassical MHC class I genes.

Previous studies have concluded that balancing selection appears to be the main mechanism that generates and maintains MHC polymorphism in vertebrates (Aguilar et al., 2004; Parham & Ohta, 1996). Positive selection is an important evidence of balancing selection. Positive selection mediated by pathogens would cause ABSs to accumulate more nonsynonymous substitutions than synonymous substitutions. Signals of positive selection were identified in *M. zibellina*. For α1 domain, positive selection acted on ABSs, which showed the nonsynonymous substitutions were significantly higher than synonymous substitutions (*d*_N_/*d*_S_ = 2.345, *p* = .034). Although the nonsynonymous substitutions at ABSs of α2 domain were higher than synonymous substitutions, it is not significant (*d*_N_/*d*_S_ = 1.187, *p* = .383). Selection pressure in α1 domain and α2 domain might often be different. Zeng et al. (2016) reported that positive selection might be more advantageous in the α1 domains of golden pheasants (*Chrysolophus pictus*). Abduriyim, Nishita, et al. (2019) reported that the nonsynonymous substitutions were significantly higher than synonymous substitutions in ABSs of α1 domain, but not in α2 domain of Eurasian badgers. However, in PAML analysis, two PSSs were identified in α2 domain. Both of the PSSs identified in α2 domain fell within ABSs. Our results suggested that both of the α1 domain and α2 domain are under positive selection pressure. We cannot deny the possibility that we misidentified a few ABSs in *M. zibellina* as they are inferred according to the study of human MHC class I (Bjorkman et al., 1987). This possibility may make the result to be more conservative as the rates of *d*_N_/*d*_S_ for non‐ABSs are usually lower than the rates of *d*_N_/*d*_S_ for ABSs. Positive selection at ABSs can permit a population or a species to present a wider repertoire of antigens, thereby enhancing the ability to  fight against pathogenic and parasitic infections. Compared with the study of the MHC class II DRB genes of *M. zibellina*, the ABSs of MHC class I genes were under stronger positive selection (Nishita, Abramov, Murakami, & Masuda, 2018), which might be explained by stronger selection pressure from intracellular pathogens than extracellular pathogens (Minias et al., 2016).

Recombination has been considered as an important factor driving evolution of MHC genes (Minias et al., 2016; Schaschl, Suchentrunk, Hammer, & Goodman, 2005). In the present study, we found that the recombination may play a role in the evolution of MHC class I genes of *M. zibellina.* The recombination signals were detected in both GARD tool and RDP4 program. The degenerate 13‐bp sequence motif CCNCCNTNNCCNC, which is essential in crossover events at human recombination hotspots (Myers, Freeman, Auton, Donnelly, & McVean, 2008), was detected at nearly half of the position of intron 2 sequences (Figure S2). We found that half of recombination breakpoints identified by RDP4 were located near the sequence motif or in the sequence motif. The intron 2 may play an important role in recombination of MHC class I genes. Although there may be some sequence structural differences between intron 2 in mammals and intron 2 in some bony fishes and anurans (Bos & Waldman, 2006; Michalova, Murray, Sultmann, & Klein, 2000; Shum et al., 2001; Zhao et al., 2013), intron 2 caused the common concern as many recombination breakpoints were found in these regions. Recombination may create new forms of ABSs. Previous studies in some species showed that the recombinant alleles observed in populations had been selectively favored (Hughes, Hughes, & Watkins, 1993). Further study of recombinant function in the future will contribute to a deeper understanding of the role of recombinant in the evolution of MHC gene. The contrasting evolutionary history between MHC class I and class II has been an interesting research topic (Kuduk, Johanet, Allaine, Cohas, & Radwan, 2012b; Minias et al., 2016). In the comparative analysis of some studies, only one of the two domains of the MHC class I gene was used for the analysis with the antigen‐binding domain of MHC class II gene. However, among MHC class I alleles, recombination is believed to tend to occur between the α1 domain and α2 domain, whereas in class II, the loop between the β‐pleated sheet and the α‐helix is suggested to be a recombination hotspot (Go et al., 2002; Jakobsen, Wilson, & Easteal, 1998). Moreover, selection pressure in α1 domain and α2 domain of MHC class I might often be different. We suggest that both α1 domain and α2 domain of MHC class I should be used for analysis of contrasting evolutionary history between MHC class I and class II.

The phylogenetic networks of α1 domain and α2 domain showed that most sequences of *M. zibellina* formed a clade with other musteline sequences (Figures 2 and 3). The clustering of the sequences among species could be due to orthology or trans‐species polymorphism. The trans‐species polymorphism is the occurrence of similar alleles in related species (Klein, Sato, Nagl, & O'HUigín, C., 1998). Without the availability of information about loci identified, it was difficult to distinguish between the trans‐species polymorphism and orthology. In addition, we found that the Mazi‐MHCI*PS01–04 clustered with the nonclassical MHC class I genes (*FLA‐S*, *Aime‐1906* and *DLA‐79*). Further study found that many carnivores all had a gene that can cluster into the clade. These genes might from a common ancestral source. We speculated that this might be a candidate nonclassical MHC class I lineage in Carnivora as the sequences of previously studied species all show the characteristics of nonclassical gene, for instance, tissue‐specific expression, abnormal exon, and limited polymorphism (Burnett et al., 1997; Hammond et al., 2012; Kuduk, Babik, et al., 2012; Zhu et al., 2012). The lineage was identified in most families of Carnivora, for instance, Felidae, Ursidae, Otariidae, Odobenidae, Phocidae, Mustelidae, Hyaenidae, and Canidae. Genome data or MHC class I studies in other families have not been reported, and we are unable to determine whether the gene is present in these families. The lineage may have preceded the divergence (about 52*–*57 Mya) of Caniformia and Feliformia (Arnason, Gullberg, Janke, & Kullberg, 2007). Though we do not know the origin of this lineage, we can know how the lineage goes through death and evolution in Carnivora. In Felidae, all the sequences are prematurely terminated in the same position in α1 domain. In Hyaenidae, the sequence of *Hyaena hyaena* became a pseudogene due to deletion within α2 domain. In Mustelidae, all the sequences are prematurely terminated in the same position. All the stop codons are located in α1 domain, but the positions of stop codons are different in Felidae and Mustelidae. We speculate that the gene had become a pseudogene before speciation. The gene was not identified in Eurasian badgers and American badger (*Taxidea taxus*), possibly due to recombination or lost. In most families of Caniformia, signals of loss of gene function were not detected. The lineage may retain its function in those families. In giant panda, *Aime‐1906* has been shown to be expressed in the liver and brain, which suggests it may be functional (Zhu et al., 2012). The strong association between *DLA‐79* and multiple immune‐mediated diseases has been demonstrated in dogs (Friedenberg et al., 2016). Though the lineage of α1 domain and α2 domain was deduced in this study, recombination might occur in other domains or introns in some species (Hammond et al., 2012). The research into the lineage could provide valuable information about the immune response mechanisms and adaptive evolution in Carnivora. Moreover, there are a large number of pseudogenes and gene fragments of MHC in the genome, which may retain important information about the evolution of MHC.

In summary, our results suggest that both balanced selection and recombination contribute to the evolution of MHC class I genes in *M. zibellina*. In addition, we identified a candidate nonclassical MHC class I lineage in Carnivora, which may contribute to further understanding of the origin and evolution of nonclassical MHC class I genes. Our study provides important immune information of MHC for *M. zibellina,* as well as other species.

## CONFLICT OF INTEREST

The authors declare that they have no competing interests.

## AUTHOR CONTRIBUTIONS

HZ, WS, BZ, and XZ designed this study. XZ, BZ, PD, LS, HD, BL, and XG performed the experiments and collected data. BZ and XZ wrote the manuscript with feedback from the authors.

## Supporting information

FigS1

FigS2

FigS3

FigS4

FigS5

TableS1

TableS2

TableS3

DataS1

Legends

## Data Availability

Data are available. DNA sequences: Genbank accessions MN274976*–*MN275001.
