# Supplementary figures and images for: Evolution of major histocompatibility complex class I genes in the sable Martes zibellina (Carnivora, Mustelidae)
Source: Ecol Evol. 2020 Mar 11;10(7):3439–49. doi: 10.1002/ece3.6140 (PMC7141072; doi:10.1002/ece3.6140)

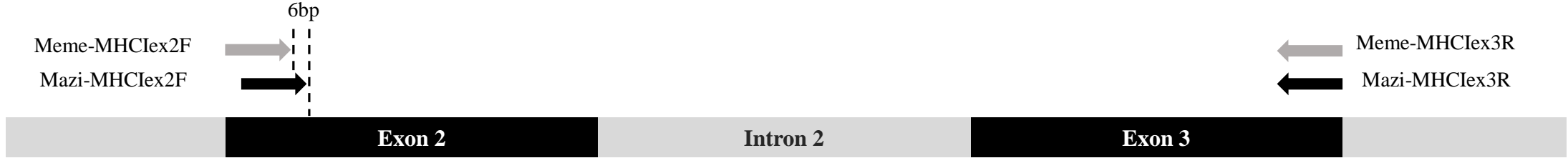

Supplement: Supplementary file 1 — FigS1 [file ECE3-10-3439-s001.pdf]

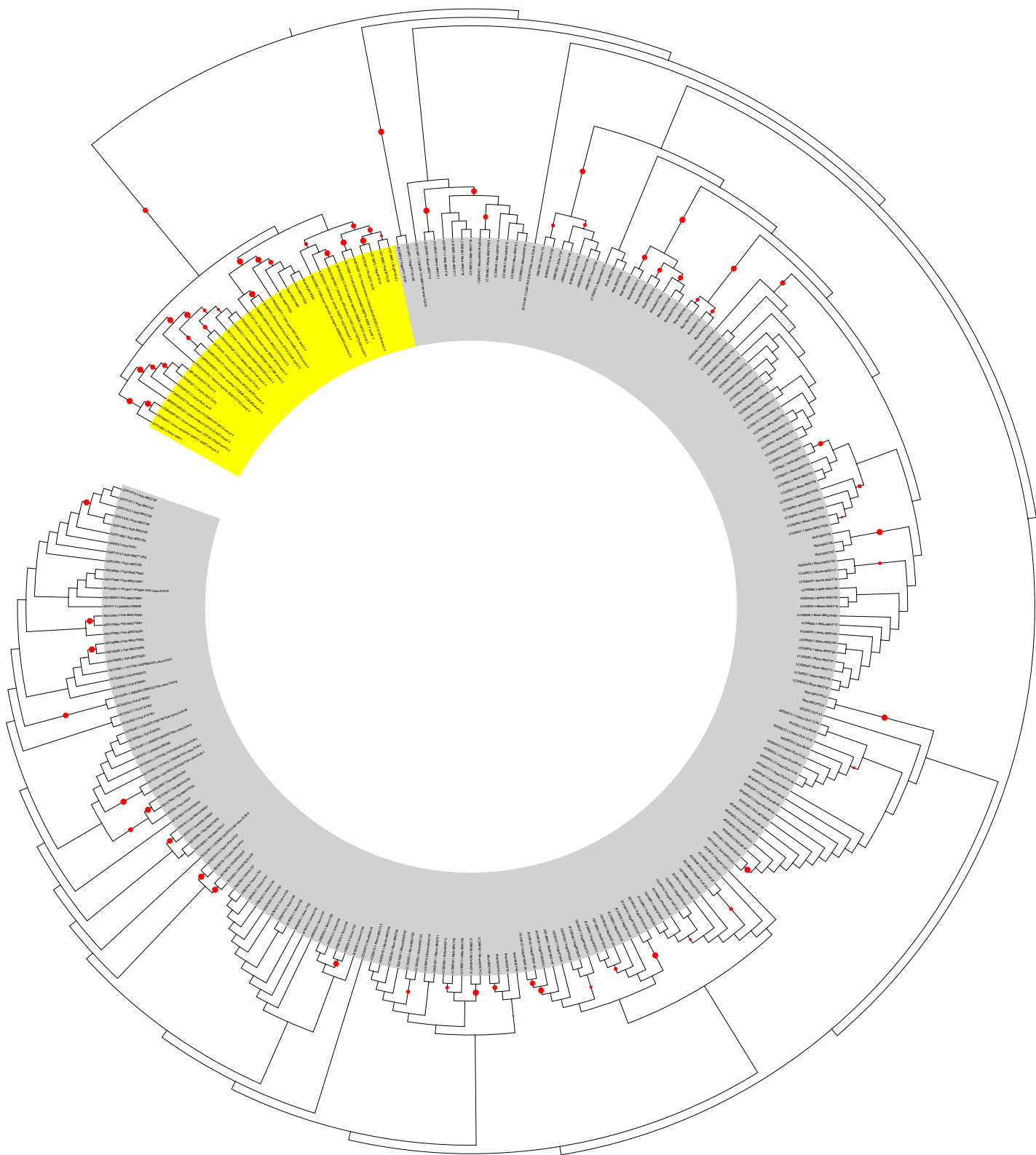

Supplement: Supplementary file 4 — FigS4 [file ECE3-10-3439-s004.pdf]

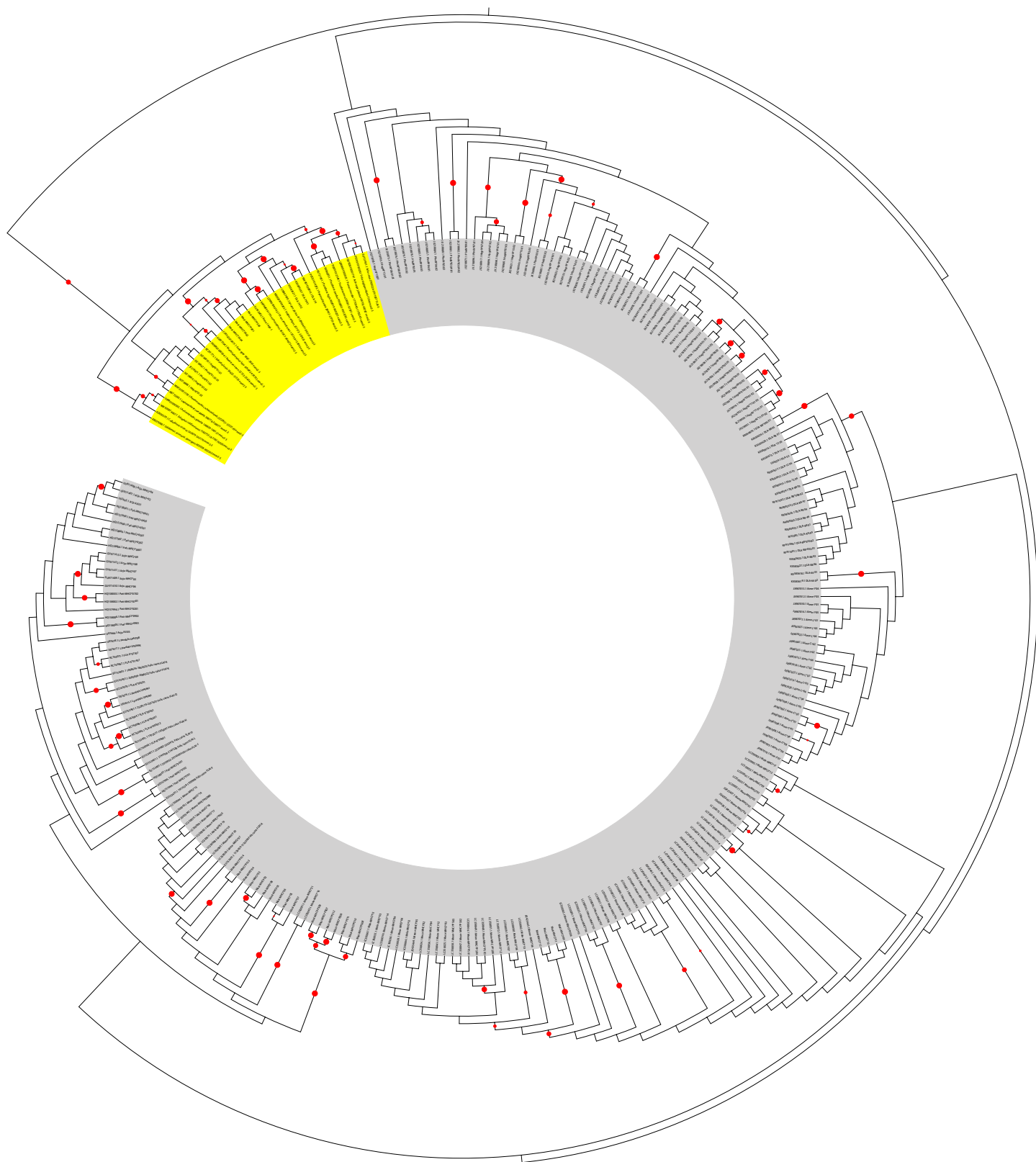

Supplement: Supplementary file 5 — FigS5 [file ECE3-10-3439-s005.pdf]
