# Supplementary material for: Evolution of major histocompatibility complex class I genes in the sable Martes zibellina (Carnivora, Mustelidae)
Source: Ecol Evol. 2020 Mar 11;10(7):3439–49. doi: 10.1002/ece3.6140 (PMC7141072; doi:10.1002/ece3.6140)
Supplement: Supplementary file 2 — FigS2 [file ECE3-10-3439-s002.pdf]

```
                   10         20         30         40         50         60         70         80         90
                                                                     #!=   #! #!  #!       #!       ! = +
Mazi-MHCI*01   YT GVSRPGRGEP RFIAVGYVDD TQFTRFDSDD ANPRDEPRAP WVEQEGPEYW DRETRICKDT AQTFRANLQT ALRYYNQSEA
Mazi-MHCI*02   .. A......... ...S...... .......... ....E....L .M........ ..N......N .....VD.N. L.........
Mazi-MHCI*03   .. A......... ...S...... .......... ....E....L .M........ ..N....... .....VD.N. M.........
Mazi-MHCI*04   H. .......... .......... .........S .SQ.Y..... .......... .......... .......... ..........
Mazi-MHCI*05   SI .......... .......... ...V.....S .SQ.M..... .......... .Q...N.... ...Y....NN LRGH......
Mazi-MHCI*06   SI .......... .......... ...V.....S .SQ.M..... .......... .Q...N.... ...Y....NN LRGH......
Mazi-MHCI*07   SI .......... .......... ...V.....S .SQ.M..... .......... .Q...N.... ...Y....NN LRGH......
Mazi-MHCI*08   .. .......... G......... ...V.....S .SR.M..... .M........ E.Q..GIR.. .....V..N. L.........
Mazi-MHCI*09   .. A......... .......... ...V.....S .SR.M..... .M........ E.Q..DL..A .....VS.NI LRG.......
Mazi-MHCI*10   .. A......... .......... ...V.....S .SR.M..... .M........ E.Q..DL..A .....VS.NI LRG.......
Mazi-MHCI*11   .. A......... .......... ...V.....S .SR.M..... .M.....K.. E.Q..DL..A .....VS.NI LRG.......
Mazi-MHCI*12   .. A......... .......... ...V.....S .SR.M..... .M.....K.. E.Q..DL..A .....VS.NI LRG.......

                       100        110        120        130        140   =    150        160        170
                                                                   = !!= !!  !  !! #!=   #!! #!  #  =
Mazi-MHCI*01   GSHTFQRMYG CDVGPDGRLL RGYSQFSYDG ADYIALNEDL RSWTAADTAA QITRRKWEAA GVAERLRNYL EVTCVEWLRR YLENG
Mazi-MHCI*02   ...NI..... .......... .....Y.... .......... .......... .......... ....Q..... ........L. .....
Mazi-MHCI*03   ...NI..... .......... .....D.... .......... .......... .......... ....Q..... ........L. .....
Mazi-MHCI*04   ......L... .......... .....Y.... .......... .......... ....S..... SE.D.Y.... ........L. .....
Mazi-MHCI*05   ....Y.S... .....H.... .....Y.... ...L...K.. .....V.AV. .......... .E.KCK.D.. .......... ...M.
Mazi-MHCI*06   ....Y.S... .....H.... .....Y.... ...L...K.. .....V.AV. .......... .E.KCK.D.. .......... ...M.
Mazi-MHCI*07   ....Y.S... .....Q.... .....Y.... ...L...K.. .....V.AV. .......... .E.KCK.D.. .......... ...M.
Mazi-MHCI*08   .......... .......... .....Y.... ......K... .......... ..S...F... ....HE.D.. .SR...S... .....
Mazi-MHCI*09   ....I..... ..M....... .....VA... ...L...... ....V..... ..S.L...E. .E..HE.... .......... .....
Mazi-MHCI*10   ....I..... ..M....... .....VA... ...L...... ....V..... ..S.L...E. .ET.HE.... .......... .....
Mazi-MHCI*11   ....I..... ..M....... .....VA... ...L...... ....V..... ..S.L...E. .E..HE.... .......... .....
Mazi-MHCI*12   ....I..... ..M....... .....VA... ...L...... ....V..... ..S.L...E. .ET.HE.... .......... .....
```
