# Supplementary material for: Evolution of major histocompatibility complex class I genes in the sable Martes zibellina (Carnivora, Mustelidae)
Source: Ecol Evol. 2020 Mar 11;10(7):3439–49. doi: 10.1002/ece3.6140 (PMC7141072; doi:10.1002/ece3.6140)
Supplement: Supplementary file 3 — FigS3 [file ECE3-10-3439-s003.pdf]

```
                    1                  20                  40                  60                  80                 100
Mazi-MHCI*01[2]     GTGAGCGACAGGGCC-GGGTCCAGGTCACGACCCCCATTCTC----------------------------CGA-TATCCGGGTCTCAGCGTCACCC-GAGTCTGGGGGACCCCTCCC
Mazi-MHCI*02[2]     .............T.-........T.................----------------------------...-......................-.......A............
Mazi-MHCI*05[3]     ...............-...........C..............----------------------------...-......................-.......A............
Mazi-MHCI*08[1]     ...............C.....AG...............C.C.ACCGCA--CCGACGGGCGGGGGTCGCCCG..G.G.........G...A......-.......A.......-...T
Mazi-MHCI*09[3]     ...............C......................C.C.ACGTTC--CTGCCAGG-----GCCTGCC.AGG.G..T.....CG....C.....T...G...C.......-----
Mazi-MHCI*11[1]     ...............C......................C.C.ACGTTC--CTGCCAGG-----GCCTGCC.AGG.G..T.....CG....C.....T...G...C.......-----
Mazi-MHCI*PS01[3]   .........GC..G.A..AGA...A...T.........C.C.AGGGACGGCGGGACGGCCGGGGTAGCCC.A.G.C....T...GG...AA.....C...G...CA....A.-----
Mazi-MHCI*PS02[1]   .........GC..G.A..AGA...A...T.........C.C.AGGGACGGCGGGACGGCCGGGGTAGCCC.A.G.C....T...GG...AA.....C...G...CA....A.-----
Mazi-MHCI*PS05[1]   ...............-..........................----------------------------...-......................C...A...A.......-....
Mazi-MHCI*PS06[6]   ...............C.............A....TG..C.C.ACAGAG--TAG---------------CC...G.G.........G..........T....A..A.......T....
Mazi-MHCI*PS11[1]   ...............C.............A....TG..C.C.ACAGAG--TAG---------------CC...G.G.........G..........T....A..A.......T....
Mazi-MHCI*PS13[1]   .....T.........C......................C.C.ACCGAC--GGGCCAGG-----ATCTCCC...G.G...T.A.GCG...A...T..C....T..A..A.A.T-----
Mazi-MHCI*PS14[1]   .....T.........C......................C.C.ACCGAC--GGGCCAGG-----ATCTCCC...G.G...T.A.GCG...A......C....T..A..A.A.T-----

                      120                 140                 160                 180                 200                 220
Mazi-MHCI*01[2]     GCACCCC---CTGTCC-CCCCCATCCTTAAGCCCGAAG-AACCCGCGGGAGCTTTTAGGCGGTTTTACTTTCTATTTGGACTTAACCCCCTGCCTGTCGGGGCGGGGCCAG-----
Mazi-MHCI*02[2]     .......---......-.....................-...............................................................T........-----
Mazi-MHCI*05[3]     .......TAC..CCGT-..........-..A...A...-...........A....C..........................................C...TC..A....-----
Mazi-MHCI*08[1]     .......CAG.CCCGT-.....................-....-......A....G.................T............................T........-----
Mazi-MHCI*09[3]     ----------------A..AGT.C..C...C..A...C-.....-.....A....C....T......T.....C...C.........A.......................-----
Mazi-MHCI*11[1]     ----------------A..AGT.C......C..A...C-.....-.....A....C....T......T.....C...C.........A.......................-----
Mazi-MHCI*PS01[3]   -----------------TG..ATG..CCC...GG...AG.....-T...GA..C.ACT.-.....GGT....AG...A.GT..T.ATGA.GATAG...CT..T...T.GG.GTCAG
Mazi-MHCI*PS02[1]   -----------------TG..ATG..CCC...GG.G.AG.....-T...GA..C.ACT.-.....GGT....AG...A.GT..T.ATGA.GATAG...CT..T...T.GG.GTCAG
Mazi-MHCI*PS05[1]   .......CAC.CC.GT-.....................-.-.........A.....-.........CG.....C...CAC.........GC.T.....CC...A..A....-----
Mazi-MHCI*PS06[6]   .......CAC.CC.GTT.....................-...........AA.....................G.G....................-.C............-----
Mazi-MHCI*PS11[1]   .......CAC.CC.GTT....-................-...........AA.....................G.G....................-.C............-----
Mazi-MHCI*PS13[1]   -----------CCCGTC...AG....CC..A..G.G..G......A....A.....G..G............C.............T.........-TC.......A....-----
Mazi-MHCI*PS14[1]   -----------CCCGTC...AG....CC..A..G.G..G......A....A.....G..G............C.............T.........-TC.......A....-----
```
