# Supplementary material for: Evolution of major histocompatibility complex class I genes in the sable Martes zibellina (Carnivora, Mustelidae)
Source: Ecol Evol. 2020 Mar 11;10(7):3439–49. doi: 10.1002/ece3.6140 (PMC7141072; doi:10.1002/ece3.6140)
Supplement: Supplementary file 7 — TableS2 [file ECE3-10-3439-s007.docx]

Table S1 Identity, frequency and distribution among individuals of MHC class I alleles detected in *Martes zibellina*

| Individual  Code | *Mazi-MHCI** | | | | | | | | | | | |  | *Mazi-MHCI*PS* | | | | | | | | | | | | | | Alleles  Detected |
| --- | --- | --- | --- | --- | --- | --- | --- | --- | --- | --- | --- | --- | --- | --- | --- | --- | --- | --- | --- | --- | --- | --- | --- | --- | --- | --- | --- | --- |
|  | 01 | 02 | 03 | 04 | 05 | 06 | 07 | 08 | 09 | 10 | 11 | 12 |  | 01 | 02 | 03 | 04 | 05 | 06 | 07 | 08 | 09 | 10 | 11 | 12 | 13 | 14 |  |
| SZ1 |  |  |  |  | + |  |  |  |  | + |  |  |  | + |  |  |  |  |  | + |  | + |  |  |  |  | + | 2(6) |
| SZ2 |  |  |  | + | + |  |  |  |  | + |  |  |  | + |  | + |  |  |  |  |  | + |  |  |  |  |  | 3(6) |
| SZ3 |  |  |  |  | + |  |  | + |  | + |  |  |  | + |  |  |  |  |  |  | + | + | + |  |  |  | + | 3(8) |
| SZ4 |  |  |  |  | + |  | + | + |  | + |  |  |  | + |  | + |  | + |  |  | + | + |  |  |  |  |  | 4(9) |
| M1 |  |  |  |  | + |  |  |  |  | + |  |  |  | + |  |  |  |  |  | + | + |  |  |  | + |  |  | 2(6) |
| M2 | + |  |  |  | + | + |  | + |  |  |  |  |  | + |  |  |  |  |  |  | + | + |  |  |  | + |  | 4(8) |
| M3 |  |  |  |  |  |  |  | + |  |  | + |  |  | + |  |  | + |  |  | + | + |  |  |  |  |  |  | 2(6) |
| M4 |  |  |  |  | + |  |  |  |  |  |  |  |  | + |  |  |  |  |  |  | + |  |  | + |  |  |  | 1(4) |
| M5 |  |  |  |  |  |  |  | + |  |  |  |  |  | + |  |  |  |  |  |  | + | + |  | + |  |  |  | 1(5) |
| M6 | + | + |  |  |  | + |  | + |  |  |  |  |  | + |  |  |  |  |  | + | + |  |  |  |  | + |  | 4(8) |
| M7 |  |  |  |  |  |  |  | + |  |  |  |  |  | + |  |  |  |  |  |  | + | + | + |  |  |  |  | 1(5) |
| M8 |  | + |  |  | + |  |  |  |  |  |  |  |  | + |  |  |  | + |  |  | + |  |  |  |  |  |  | 2(5) |
| G1 |  |  |  |  |  |  |  |  | + | + | + |  |  |  |  |  |  | + | + |  |  | + |  |  |  |  |  | 3(6) |
| G2 |  |  |  |  | + |  |  |  |  |  | + |  |  |  | + |  |  | + |  |  |  | + |  |  |  |  |  | 2(5) |
| G3 |  |  |  |  | + |  |  |  | + | + | + | + |  |  |  |  |  | + | + |  |  |  |  |  | + |  |  | 5(8) |
| G4 |  |  |  | + | + |  |  |  |  |  |  |  |  |  | + |  |  |  |  |  |  | + |  |  |  |  |  | 2(4) |
| G5 |  |  |  |  | + |  |  |  |  |  |  |  |  | + |  |  |  |  |  |  | + | + |  |  |  |  |  | 1(4) |
| G6 |  |  | + | + | + |  |  | + |  |  |  |  |  | + |  |  |  |  |  |  | + |  |  |  |  |  |  | 4(6) |
| G7 |  |  |  | + | + |  |  | + |  |  |  |  |  | + |  |  |  |  |  |  | + | + |  |  |  |  |  | 3(6) |
| G8 |  |  |  | + |  |  |  | + |  |  |  |  |  | + |  |  |  |  |  |  | + | + |  |  |  |  |  | 2(5) |
| G9-1 |  |  |  | + |  |  |  | + |  |  |  |  |  | + |  |  |  |  |  |  |  | + |  |  |  |  |  | 2(4) |
| G10-2 |  |  |  | + |  |  |  | + |  |  |  |  |  | + |  |  | + |  |  |  |  | + |  |  |  |  |  | 2(5) |
| G11-3 |  |  |  |  | + |  |  | + |  |  |  |  |  | + | + |  |  |  |  | + |  |  |  |  |  |  |  | 2(5) |
| T1 |  |  |  | + | + |  |  |  |  |  |  |  |  | + |  |  |  |  |  |  | + | + |  |  |  |  |  | 2(5) |
| Total | 2 | 2 | 1 | 8 | 16 | 2 | 1 | 13 | 2 | 7 | 4 | 1 |  | 20 | 3 | 2 | 2 | 5 | 2 | 5 | 15 | 16 | 2 | 2 | 2 | 2 | 2 | 59(139) |

In the“Individual Code” column, SZ indicates Shangzhi, M indicates Mudanjiang, G indicates Genhe, T indicates Tahe. In the “Alleles Detected” column, the first value omits pseudogenes, the value in the parentheses includes pseudogenes.
