# Supplementary material for: Evolution of major histocompatibility complex class I genes in the sable Martes zibellina (Carnivora, Mustelidae)
Source: Ecol Evol. 2020 Mar 11;10(7):3439–49. doi: 10.1002/ece3.6140 (PMC7141072; doi:10.1002/ece3.6140)
Supplement: Supplementary file 8 — TableS3 [file ECE3-10-3439-s008.docx]

Table 2 Recombinants detected in *Martes zibellina* MHC class I alleles; their parent sequences and breakpoints (BP) identified by the RDP and the GARD; and the results of RDP analyses

|  |  | Recombination event 1 | Recombination event 2 |
| --- | --- | --- | --- |
|  | Recombinant | *Mazi-MHCI*08* | *Mazi-MHCI*02* |
|  | Major Parent | *Mazi-MHCI*02* | *Mazi-MHCI*07* |
|  | Minor Parent | *Mazi-MHCI*11* | Unkonwn (*Mazi-MHCI*11*) |
|  | Breakpoint 1 location | 42 (42) | 280 (279); *P* < 0.001 |
|  | Breakpoint 2 location | 322 (322) | 660 (628); *P* < 0.05 |
| RDP methods | RDP | NS | <0.05 |
|  | GENECONV | <0.001 | <0.001 |
|  | BootScan | <0.01 | <0.05 |
|  | MaxChi | <0.001 | <0.01 |
|  | Chimaera | <0.01 | <0.01 |
|  | SiScan | <0.001 | <0.001 |
|  | 3Seq | <0.001 | <0.01 |
|  | BP from GARD | 148^**^ | |

NS indicates not significant. The numbers in parentheses are BP locations in the recombinants nucleotide sequences without gaps. The values after the semicolon are MaxChi values for that BPs. ** indicate *P* < 0.01.
