# Supplementary material for: Evolution of major histocompatibility complex class I genes in the sable Martes zibellina (Carnivora, Mustelidae)
Source: Ecol Evol. 2020 Mar 11;10(7):3439–49. doi: 10.1002/ece3.6140 (PMC7141072; doi:10.1002/ece3.6140)
Supplement: Supplementary file 9 — DataS1 [file ECE3-10-3439-s009.docx]

>CM007430.1_Lycaon_pictus_41129604-41130313_exon2-3

AACACCGTCGTATCCCGGCCTGGCCACGGGGAGCCCCGCCACTGGGCAGTCGCCTACGTGGACGACACACCGTTCGAACGGTTCGACAGCGAACGCGAGAGTCGGCGGCCTGAGCCGCTGGTGCGGTGGCTGGAGCAGGAGGGGCCTGAGTACTGGGAAGAGCGCACGCTGAACTCCAGGACCTGCACACAGGTCCTCCGAGGGACGCTGAACGAAGTTTCGCAGGACTACTACAATCAGAGCAGGACCGGTGAGCCACGGGGGCGGGGCCAGGTCGCCATCCCCGTCTCCGCCCACGGGCCGAGGTCGCCCCGAGTGTCTGGGGCCGAGGGCCACCCCGAGGCTGCGGGACTCACCCGTCCCCGTCCCCGAAAGGGAAGGAGACTCCTGGGGACGCGACTGGGTTTTGTTTTCAGTTGTAGACTTTCGTGCTGACACATCGTGGGGCGGGGCCAGGGTCTCACACCTTCCAGACTATAACAGGCTGCGAAGTGGGACCTGATGGGCGCTTCCTGCGCGGGTACCAGCGTAACGCCTATGACGGCCTGGATTACATCACCCTGAGCGAGGACCTGAGCTCCTGGATCGTGGAGGACCCGGTGGCTCAGATCACTCTGCGCAAGTGGGATGCGGCCAGTGTAGCTGAGAACAATAAGAACTTCCTGGAGGGCAGGTGCTTGGAGTGGCTCCGCAGACACCTGGAGAATGGG

>CYRY02010071.1_Gulo_gulo_1887-2601_exon2-3

TTCACTGTGGTGTCCCAGCCTAGCCGCGGGGAGCCCCGGTATTTGGAACTCAGCTACGTGGACGACACGCAGTTGGCGTGGTTTGACAGCGACTCTGCCAGTCTGAGGATGGAGCCGCGGGTGCCGTGGGTGGAGCAGGTGGGGCTGGAGTATTGGGACCAGCAGACCCGGGACTCCAGGACGTGCACACAGACACTCTGAGCGAGGCTGCGAGAAGTGGGCGGCTACTACGACCAGAGCGAGGCCGGTGAGCGACGCGGGCAGGAGACAGATCATGACCCCCATCCCCAGGGACCGCGGGACGGCCGCGGGACGGCCGGGGTAGCCCCAAGTCTCCGTGTCGGAGCGACACCCCGAGGCTGCGGGACACTGCCATGCCCCCAGCGGGGAAGAACCCTGGGGACTCTACTGGGTTTGGTTTTCAGTTTAGGTTTTAATGACGATAGGTCCTGGTGGGTCAGGGTTTCACACCTTCCAGAGAGTGATAGGCTGCGACGTGGGGCCTGATGGGCGCTTTCTGCGCGGGTACCTTCGTAATGCCTATGATGGCATGGATCACATCGCCCTGAACAAGGCCCTGTGCTCCTGGACCGAGGCAGACTCCACAGCGCAGATCACCCGTCAGAAGTGGGAGGCGACTGGTGTGGCAGAGCGCTACAGGAAATACATGGAGGGCAGATGCGTGGAGTGGCTCCACAGGCACCTGGAGAAGGGG

>FNWR01000504.1_Neovison_vison_22723-23436_exon2-3

TTCACTGTGGTGTCCCAGCCTGGCCGCAGGGAGCCCCGGTATTTGGAACGCAGCTATGTGGACGACACGCAGTTCCCGCTGTTTGACAGCGACTCTGCCAGTCTGAGGATGGAGCCACGGGCGCCCTGGGTGGAGCAGGAGGGGCTGGAGTATTGGGCCCAGCAGACCCGGGACTCTAGGACATGCATACAGACACTCTGAGGGAGGCTGCAAGAAGTGGGCAGCTACTGCAACCCAAGCGAGGCCGGTGAGCGACGCGAGCAGGAGACAGATCATGACCCCCATCCCCAGGGACCGCCGGATGGCTGGCCGGGGTAGCTCCAAGTCTCCATGTTGGAGCGACACCCCGAGGCTGCGGGGCACCGCCATGCCCCCAGCGGGGAAGAATCCTGGGGACTCTACTGGGTTTGGTTTTCAGTTTAGGTTTTAATGACAATAGGTCCTGGTGGGCCAGGGTCAGGGTTTCACACCTTCCAGAGAGTGATAGGCTGCCACGTGGGGCCTGATGGGCGCTTTCTGCACGGGTACCTTCGTGATGCCTATGATGGCGTGGATCACATCGCCCTGAATAAGGACCTGTGCTCCTGGACCAAGGCAGACTCCACCGCGCAGATCACTGGTCAGAAGTGGGAGGCAACTGGTGTGGCAGAGCAGTACAGGAACTATATGGAGCGCAGATGCATGGAGTGGCTCCACAGGCACATGGAGAAGGGG

>GL897304.1_Mustela_putorius_furo_479482-478792_exon2-3

TTCACTGTGGTGTCCCAGCCTGGCCGCAGGGAGCCCCGGTATTTGGAACACAGCTATGTGGACGACATGCAGTTCCCGCTGTTTGACAGCGACTCTGCCAGTCTGAGGATGGAGCCGGGGGCGCCCTGGGTGGAGCAGGAGGGGCTGGAGTATTGGGCCCAGCAGACCCGGGACTCCAGGACGTGCACACAGACACTCTGAGGGAGGCTGCGAGAAGTGGGCAGCTACTGCAACCCGAGCGAGGCCGGTGAGCGACACGGGCAGGAGACAGACCATGACCTCCATTCCCAGGGACCGCCGGATGGCCGGCCGGGGTAGCTCCAAGTCTCCGTGTTGGAGCGACACCCCGATGCTGCGGGGCACCGCCATCCCCCCAGCGGGGAAGAACCCTGGGTACTCTACTGGTTTTGGATTTAATGACAATGGGCCGGGGTCAGGGTTTCACACCTTCCAGAGAGTGATAGGCTGCGACGTGGGGCCTGATGGGCGCTTTCTGCGCGGGTACCTTCATGATGCCTATGATGGCATGGATCACATCGCCCTGAATAAGGACCTGTGCTCCTGGACCAAGGCAGACTCCACCGCACAGATCACTGGTCAGAAGTGGGAGGCGACTGGTGTGACAGAGCAGTACAGGAACTACGTGGAGCACAGATGCGTGGAGTGGCTCCACAGGCACCTGGAGAAGGGG

>KB229288.1_603028-602343_Odobenus_rosmarus_divergens_exon2-3

CTCACCGTCACGTCCCGGCCCGGCCGCGGGGAGCCCCGGTACGTGGAAGTCGGCTACGTGGACGACACGCAGTTCGCGCGGTTCGACAGCGACTCTGTCAGTCTGAGGATGGAGCCGCGGGCGCCGTGGGGGGGGCAGGAGGGGCCGGAGTACTGGGACCGGCTGACGCGGGACTCCAGGACGTGCACACAGACTCTCCGAGGGAAGCTGAACGAAGTGCGCGGCTACTACAACCAGAGCGAGGCAGGTGAGCGACCGGGCAGGGGACAGATCACGATCCCCATCCCCACGGACGGGCCGGGGTCGCCCCAAGTCTCCGTGTCCGAGCACCACCCCGAGGCTGCGGGACACCGCCGTACCCCGAACAGGGAAGAACCGTTTGGTTTTCAGTTTAGGCTTTAATGACGATAGGTCGTGGTGGGCCGGGGCCAGGGTCTCACACCATCCAGAGAATGTACGGCTGCGACGTGGGGCCTGATGGGCGCTTCCTGCGCGGGTACTTTCGTGAGGCCTACGACGGCGCGGATTACATCGCCCTGAACGAGGACCTGCGCTCCTGGACCGCGGCGGACACGACGGCGCAGATCACCCGGCGCAAGTGGGAGGCGGCCGGTGCGGCGGAGCGGTGGCGAAACTACCTGGAGGGCACCTGCGTGGAGTGGCTCGGCAGGCACCTGGAGCACGGG

>KB715260.1_Leptonychotes_weddellii_308778-308077_exon2-3

TTCACTGTCGTGTCCCGGCCCGGCCGCGGGGAGCCCCGGTACGTGGAAGTCTGCTACGTGGACGACGTGCAGTTCGCGCGGTTCGACAGCGACGCGGCGAGTCGGAGGATGGAGCCGCGGGCGCGGTGGGTGGAGCAGGAGCGGCCGGAGTACTGGGCCCAGCAGACGCGGGACTCCAGGACGTGCACACAGACTCTCCGAGGGAATCTGAACGAAGCGCGCGGCTACTACAACCAGAGCGAGGTAGGTGAGCGACGCGGGCAGGGGACAGATCACGATCCCCATCCCCCCGGACGGCCAGGGTCGCCCCAAGTCTCCGTGTCCGAGCGACACCCCGAGGCTGCGGGACACCGCCGTCCCCCGAACGGGGAAGAACCCTTGGGGAGTCTACTCGGTTTGGTTTTCAGTTTAGGCTTTAATGACGATAGGTCGTGGTGGGCCGGGGCCAGGGTCTCATACCTTCCAGAGAATGATAGGGGGCGACGTGGGCCTGATGGGCGCTTCCTGCTTGGGTACCTTCGTGATGCCTACGACGGCGCGGATTACATCGCCCTGAACGAGGACCTGCGCTCCTGGACCGCGGCGGGCACGGCGGCGCAGATCACCCGGCGCAGGTGGGAGGCGGCCGGTGTGGCAGAGCGCTCCAGGAACTATCTGGAGGGCACCTGCGTGGAGTGGCTCGGCAGGTACCTGGAGAACGGG

>KE721931.1_Panthera_tigris_809919-809235_exon2-3

CACACCGCGGTGTCCCGGCCGGGCCGCGGGGAGCACGGGTACTTGGAAGTCGGCTACGTGGACGACACGCAGTTCGTGCGGTTCGACAGAGACACCCCGAGTTCGGTGATGGAGCCGCGGTTGCAGTGGGTGGACCAGGAGGGGCCGGAGTAGTGGGAGCAGCAGACGCAGAACTTCAGGACCCGCACACAAACTCTTCAAGTGAGGCTGAATGAATTGCGTGTCCACTACAGCCAGAGCAAGGCCGGTGAGCGACCTGGGTCCGGGTCCAGGTCACGACCCCCGTCCCTACGGACGCGCCAAGGTCGCCCCGAGTGTCTGGGTCCTAGTGCCACGCGGAGTCTGCGCCCCACCCCCGTCCTGCGAATAGGTAAGAGCCCCTGGGGACTGTACTCGGTTTGGTTTTCAGTTTAGGCTTTAATGATTGCAGGTCAGGGCGGGGCCAGTGTCTCACACCTTCCAAAGGATGACAGGCTGCGACGTGGGGCCTGATGGGCACTTCCTCTGCCGTTACCTTCGTGACACCTACGACCGCGCGGATTACATCACCCTGCGGGTGCCATGGCGGGACACCGCGCCGCACATCACCCGCTGCAAGTGGGAGGCAGCCGGTGAGGCGGAGCGCTACAGGTACTACCTGGAGGGCACGTGCATGGAGTGGTTCCTCAAGTATCTGGAGATGGGG

>KK498659.1_Ursus_maritimus_563977-564673_exon2-3

CGCACGGTCATATCCCGACCTGGCAGCGCGGAGCCCCGGTACTTGGAGGTCGGCTATGTGGACGACGCGCACTTCGTGCGGTTCGACAGCGACGGCGCGAGTCCGAGGGCTGAGCTGCGGGCGCCGTGGTTGGAGCAGGAGGGGCCGGAGTTTTGGGACTGGAAGACGCAGAACACCAGGACCTGCACACACATTCTCCGAGGGAGGCTGAACGAAGTGCGCGGCTACTACAACCAGAGCGAGGAAGGTGAGCGACGTGGGCTCGGGCCCAGGTCACGATCCCCACTCTCACGGACGGGCCGAGGTCACCCCGAATGTCTGGGGCCAAGAGCCACCCCGAGGCTGCGGTTTTCACCTGTCCTCAGAGCAGGGAAGAGCCGCTGGGGCCGCTACTGGGTTTTAAGTTTAGGCTTTCATGATGACCCGTGGTGGGGCGGGGCCAGGGTCTCACACCTTCCAGAGTATGATTGGCTGCGACATGGGGCCTGATGGACGCTTCCTTCGCGGGTACTTTCGTAACGCCTATGATGGCGCGGATTACATCACCCTGAATGAGGACCTGCGCTCCTGGACCGCGGCGAACTCAGAGGCGCAGATCACCAGACGCATGTGGGAGGCATCTGGTGTAGCCGAGAGGAGTAGGAACTACCTGGAGGGCTCGTGCATGGAGTGGCTCCGCAGGCACCTGAAGAATGGG

>KV860351.1_Panthera_pardus_9950601-9951285_exon2-3

CACACCGCGGTGTCCCGGCCGGGCCGCGGGGAGCCCGGGTACTTGGAAGTCGGCTACGTGGACGACACGCAGTTCGTGCGGTTCGACAGAGACACCCCGAGTTCGGTGATGGAGCCGCGGTTGCAGTGGGTGGACCAGGAGGGGCCGGAGTAGTGGGAGCAGCAGACGCAGAACTTCAGGACCCGCACACAAACTCTTCAAGTGAGGCTGAATGAATTGCGTGTCCACTACAGCCAGAGCAAGGCCGGTGAGCGACCTGGGCCCGGGTCCAGGTCACGACCCCCGTCCCTCCGGACGCGCCAAGGTCGCCCCGAGTGTCTGGGTCCTAGTGCCACGCGGAGTCTGCGCCCCACCCCCGTCCTGCGAATAGGTAAGAGCCCCTGGGGACTGTACTCGGTTTGGTTTTCAGTTTAGGCTTTAATGATTGCAGGTCAGGGCGGGGCCAGTGTCTCACACCTTCCAAAGGATGACAGGCTGCGACGTGGGGCCTGATGGGCACTTCCTCTGCCGTTACCTTCGTGACACCTACGACCGCGCGGATTACATCACCCTGCGGGTGCCATGGCGGGACACCGCGCCGCACATCACCCGCTGCAAGTGGGAGGCAGCCGGTGAGGCGGAGCGCTACAGGTACTACCTGGAGGGCACGTGCATGGAGTGGTTCCTCAAGTATCTGGAGATGGGG

>KZ291775.1_Enhydra_lutris_50622-51335_exon2-3

TTCACTGTGGTGTCCCGGCCTGGCCGCGGGGAGCCCCGGTATTTGGAACGCAGCTACGTGGACGACACGCAGTTCGCGCAGTTTGACAGCGACTCTGCCAGTCTGAAGATGGAGCCGCGGGCACCGTGGGTGGAGCAGGAGGGGCTGGAGTATTGGGACCAGCAGACCCGGGACTCCAGGACGTGCACACAGACACTCTAAGGGAGGCTGGGAGAAGTGGGCGGCTACTACAACCAGAGCGAGGCCGGTGAGCGACAGGGGCAGGAGACAGATCATGACCCCCATCCCCAGGGACGGCCGGACGGCCGGCTGGGGTAGCTCCAAGTCTCCGTGTCGGAGCAACACCCCGAGGCTGCGGTCCACCGCCATGCCCCCAACGGGGAAGAACCCTGGGGACTCTACTGGGTTTGGTTTTCAGTTTAGGTTTTAATGACAATAGGTCCTGGTGGGCCGGGGTCAGGGCTTCACACCTTCCAGAGAGTGATAGGCTGCGACGTGGGGCCTGATGGGCGCTTTCTGCGCGGGTACCTTCGTGATGCCTATGATGGCGTGGATCACATCGCCCTGAATAAGGACCTGTGCTCCTGGACCGAGGCAGACTCCACCGCGCAGATCACCCGTCTGAAGTGGGAGGCGACTGGTGTGGCCCAGCGGTACAGGAACTACATGGAGGGCAGATGCATGGAGTGGCTCCACAGGCACCTGGAGAAGGGG

>KZ836329.1_Callorhinus_ursinus_542879-542176_exon2-3

CTCACTGTCACGTCCCGGCCCGGCCGCGGGGAGCCCCGGTACGTGGAAGTCGGCTACGTGGACGACACGCAGTTCGCGCGGTTCGACAGCGACTCTGTCAGTCTGAGGTACGAGCCGCGGGCGCCGTGGAGGGGGCGGGAGGGGCCGGAGTACTGGAACCGGCTGACGCGGAACGCCAAGGACAGCGCACAGACTCTCCGAGGGAAGCTGAACGAAGTGCGCGGCTACTACAACCAGAGCGAGGCAGGTGAGCGACGCGGGCAGGGGACAGATCACGATCCCCATCCCCACGGACGGGCCGGGGTCGCCCCAAGTCTCCGTGTCCGAGCACCACCCCGAGGCTGCGGGACACCGCCGTACCCCGAACGGGGAAGAACCCTTGCGGAGTCTACTGGGTTTGGTTTTCAGTTTAGGCTTTAATGACGATAGGTCGTGGTGGGGCGGGGTCAGGGTCTCACACCATCCAGAGAATGTACGGCTGCGACGTGGGGCCTGATGGGCGCTTCCTGCGCGGGTACTTTCGTGAGGCCTACGACGGCGCGGATTACATCGCCCTGAACGAGGACCTGCGCTCCTGGACCGCGGCGGACACGACGGCGCAGATCACCCGCCGCAAGTGGGAGGCGGCCGATGCGGCGGAGCGGTGGCGAAACTACCTGGAGGGCACCTGCGTGGAGTCCCTGCGCAGGCACCTGGAGCACGGG

>LZNR01005162.1_Ursus_americanus_153750-153054_exon2-3

CGCACGGTCATATCCCGACCTGGCAGCGCGGAGCCCCGGTACTTGGAGGTCGGCTATGTGGACGACGCGCACTTCGTGCGGTTCGACAGCGACGGCGCGAGTCCGAGGGCTGAGCTGCGGGCGCCGTGGTTGGAGCAGGAGGGGCCGGAGTTTTGGGACTGGAAGACGCAGAACACCAGGACCTGCACACACATTCTCCGAGGGAGGCTGAACGAAGTGCGCGGCTACTACAACCAGAGCGAGGAAGGTGAGCGACGTGGGCTCGGGCCCAGGCCACGATCCCCACTCCCACTGACGGGCCGAGGTCACCCCGAATGTCTGGGGCCAAGAGCCACCCCGAGGCTGCGGTTTTCACCTGTCCCCAGAGCAGGGAAGAGCCGCTGGGGCCGCTACTGGGTTTTAAGTTTAGGCTTTCATGATGACCCGTGGTGGGGCGGGGCCAGGGTCTCACACCTTCCAGAGTATGATTGGCTGCGACATGGGGCCTGATGGACGCTTCCTTCGCGGGTACTTTCGTAACGCCTATGATGGCGCGGATTACATCACCCTGAATGAGGACCTGCGCTCCTGGACCGCGGCGAACTCAGAGGCGCAGATCACCAGACGCATGTGGGAGGCATCTGGTGTAGCCGAGAGGAGTAGGAACTACCTGGAGGGCTCGTGCATGGAGTGGCTCCGCAGGCACCTGAAGAATGGG

>NBDQ01000303.1_Vulpes_vulpes237172-237872_exon2-3

AACACCGTCGTATCCCGGCCTGGCCACGGGGAGCCCCGCCACTGGGGAGTCGCCTACGTGGACGACACGCCGTTCGAGCGGTTCGACAGCGAACGCGAGAGTCGGCGGCCGGAGCCGCTGGTGCGGTGGCTGGAGCAGGAGGGGCCTGAGTACTGGGAGGAGCGCACGCTGGACTCCAGGACCTGCACACAGGTGCTCCGAAGGAGGCTGAATGAGGCGCACGGCTACGACAATCAGAGCAGGACCGGTGAGCCACGGGGGCGGGTGCAGGTCACCATCCCGGTCTCCGCCCACGGGCCGAGGTAGCCCCGAGCGCCTGGGGCCGAGTGCCACCCCGAGGCTGCGGGACTCACCCGTCCCCGAAAGGGAAAGAGACTCCTGGAGACGCTACTGGGTTCTGTTTTCAGTTGTAGGCTTTCGTGCTGACACATCGTGGGGCGGGGCCAGGGTCTCACACCTTCCAGACTATAATAGGCTGCGACGTGGGACCTGATGGGCGCTTCCTGCGCGGGTACCAGCGTAACGCCTACGACGGCCTGGATTACATCACCCTGAACGAGGACCTGAGCTCCTGGATCGTGGAGGACCCGGTGGCGCAGATCACTCTGCGCAAGTGGGAGATGGCCGGTGTAGCTGAGAACAATAAGAACTTCCTGGAGGGCAGGTGCTTGGAGTGGCTCCGCAGACACCTGGAGAATGGG

>NINY01007739.1_Neomonachus_schauinslandi_2252851-2252149_exon2-3

TTCACTGTCGTGTCCCGGCCCGGCCGCGGGGAGCCCAGGTACGTGGAAGTCAGCTACGTGGACGACGTGCAGTTCGCGCGGTTCGACAGCGACGCGGCAAGTCGGAGGATGGAGCCGCGGGCGCGGTGGGTGGAGCTGGAGCGGCGGGAGTACCCGGCCCAGCAGACGCGGGACTCCAGGACTTGCACACAGACTCTCCGAGGGAATCTGAACGAAGCGCGCGGCTACTACAACCAGAGCGAGGTAGGTGAGCGACGCGGGCAGGGGACAGATCACGATCCCCATCCCCCCGGACGGCCAGGGTCGCCCCAAGTCTCCGTGTCCGAGCGACACCCCGAGGCTGCGGGACACCGCCGTCCCCCGAACGGGGAAGAACCCTTGGGGAGTCTACTCGGTTTGGTTTTCAGTTTAGGCTTTAATGACAATAGGTCGTGGTGGGCCGGGGCCAGGGTGTCATACCTTCCAGAGAATGATAGGCTGCGACGTGGGGCCTGATGGGCGCTTCCTGCTTGGGTACCTTCGTGATGCCTACGACGGCGCGGATTACATCGCCCTGAACGAGGACCTGCGCTCCTGGACCGCGGCGGACACGGCGGCGCAGATCACCCGGCGCAAGTGGGAGGCGGCCGGTGTGGCAGAGGACTTGAGGAACTACCTGGAGGGCACCTGCGTGGAGTGGCTCGGCAGGCACCTGGAGAACGGG

>NW_020874467.1_Zalophus_californianus_118275318-118276020_exon2-3

TCACTGTCACGTCCCGGCCCGGCCGCGGGGAGCCCCGGTACGTGGAAGTCGGCTACGTGGACGACACGCAGTTCGCGCGGTTCGACAGCGACACTGTCAGTCTGAGGTACGAGCCGCGGGCGCCGTGGATCGAGCGGGAGGGGCCGGGGTACTGGGACCGGCAGACGCGAGGCTCCAGGAATTGCGTACAGACTCTCCGAGGGAAGCTGAACGAAGTGCGCGGCTACTACAACCAGAGCGAGGCAGGTGAGCGACGCGGGCAGGGGACAGATCACGATCCCCATCCCCACGGACGGGCCGGGGTCGCCCCAAGTCTCCGTGTCCGAGCACCACCCCGAGGCTGCGGGACACTGCCGTACCCCGAACGGGGAAGAACCCTTGCGGAGTCTACTGGGTTTGGTTTTCAGTTTAGGCTTTAATGACGATAGGTCGTGGTGGGGCGGGGCCAGGGTCTCACACCATCCAGAGAATGTACGGCTGCGACGTGGGGCCTGATGGGCGCTTCCTGCGCGGGTACTTTCGTGAGGCCTACGACGGCGCGGATTACATCGCCCTGAACGAGGACCTGCGCTCCTGGACCGCGGCGGACACGGCGGCGCAGATCACCCGCCGCAAGTGGAAGGCGGCCGGTGAGGCGGAGCGGTGGCGAAACTACCTGGAGGGCACCTGCGTGGAGTCCCTGCGCAGGCACCTGGAGCACGGG

>PISV01012544.1_Panthera_onca_58081-87397_exon2-3

CACACCGCGGTGTCCCGGCCGGGCCGCGGGGAGCCCGGGTACTTGGAAGTCGGCTACGTGGACGACACGCAGTTCGTGCGGTTCGACAGAGACACCCCGAGTTCGGTGATGGAGCCGCGGTTGCAGTGGGTGGACCAGGAGGGGCCGGAGTAGTGGGAGCAGCAGACGCAGAACTTCAGGACCCGCACACAAACTCTTCAAGTGAGGCTGAATGAATTGCGTGTCCACTACAGCCAGAGCAAGGCCGGTGAGCGACCTGGGCCCGGGTCCAGGTCACGACCCCCGTCCCTACGGACGCGCCAAGGTCGCCCCGAGTGTCTGGGTCCTAGTGCCACGCGGAGTCTGCGCCCCACCCCCGTCCTGCGAATAGGTAAGAGCCCCTGGGGACTGTACTCGGTTTGGTTTTCAGTTTAGGCTTTAATGATTGCAGGTCAGGGTGGGGCCAGTGTCTCACACCTTCCAAAGGATGACAGGCTGCGACGTGGGGCCTGATGGGCACTTCCTCTGCCGTTACCTTCGTGACACCTACGACCGCGCGGATTACATCACCCTGCGGGTGCCATGGCGGGACACCGCGCCGCACATCACCCGCTGCAAGTGGGAGGCAGCCGGTGAGGCGGAGCGCTACAGGTACTACCTGGAGGGCACGTGCATGGAGTGGTTCCTCAAGTATCTGGAGATGGGG

>PISY01018928.1_Felis_nigripes_6844-7544_exon2-3

CACACCGCGGTGTCCCGGCCGGGCCGCGGGGAGCCCGGGTACTTGGAAGTCGGCTACGTGGACGACAAGCAGTTCGTGCGGTTCGACAGCGACACCCCGAGTTCGGTGATGGAGCCGCGGTTGCAGTGGGTGGACCAGGAGGGGCCGGAGTAGTGGGAGCAGCAGACGCAGAACTTCAGGACCCGCACACAAACTCTTCAAGTGAGGCTGAATGAATTGCGTGTCCACGACAGCCAGAGCAAGGCCGGTGAGCGACCTGGGCCCGGGTCCAGGTCACGACCCCCGTCCCTACGGACGCGCCAAGGTCGCCCTGAGTGTCTGGGTCCTAGTGCCACGCTGAGTCTGCGCCCCACCCCCGTCCTGCGAATAGGTAAGAGCCCCTGGGGACTGTACTCGGTTTGGTTTTCAGTTTAGGCTTTAATGATTTCAGGGCAGGGCAGGGCCAGTGTCTCACACCTTCCAAAGGATGATAGGCTGCGACGTGGGGCCTGATGGGCACTGCCTCCGCCGTTACCTTCGTGAGGCCCACGACCGCGCGGATTACATCACCCTGAACCAGGACCTGCGCTCCTGGACCATGGCGGGACACCGCGCCGCACATCACCCGCTGCAAGTGGGAGGCAGCCGGTGAGGCGGAGCGCTACAGGTACTACCTGGAGGGCACGTGCATGGAGTGGTTCCTCAAGTATCTGGAGATGGGG

>PITC01019292.1_Hyaena_hyaena_28497-27750_exon2-3

CACACCACAGTGTCCCAGCCTGGCCATGGGGAACCCCGCTTCATCTCTGTGGGCTACGTGGATGACACTTGGTTCATGCAGTTTGACAGCGATGCCCCGAGTTCGAGAATGGAGCCGCGGTCGCTGTGGGTGGAGCAAGAAGGGCCCGAGTATTGGGAGCAGCAGACGCAGGACTTCAGGACCTACACACAGACTCTTCAAGTGAGGCTGAACGAATTGAGTGACCACTACAGCCAAAGCAAGGCCGGTGAGTGACGTGTGTCCGGGTCCAGGTCATGAACCCCGTCCTCACAGACGCGCTGAGGTCGCCCCGAGTGTCTGGGTCCTAGCGTCACGCCGAATCTACGGAGCGCCCCCCCCCCCCCCCCCCCGCTCCCTCACCTGTCCCCAGGTCCTGTAGGTCCTGTGAATGGGGAAGAAGCCCTGGGAACTGTATTTGGTTTGGTTTTCAGTTTAGGCTTTAAAGACTGCAGCTCGGGCTGGGGGCGGGGCCAGGGTCTCACACCTTCCAAAGGATGATAGGCTGCAACCTGGGGCCTGACGGGCACTTCTTCCACGGGACCTTCGTGAAGCCTACGACGGCGCGGACTACATAATCCTGAACAACGACCTACGCTCCTGGACCGCGTCAGACACAGCGGCGCAGATCAGCCGCCGCAAGTGGGAGGTGGCAGGTGAGGCGGAGCGCTTCAGGAATTACCTGGAAGGCACTTGCTTGGAGTGGCTCCTCAGGCACCTGGAGAACTGG

>QAVW01000931.1_Puma_concolor_1770940-1770240_exon2-3

CACACCGCGGTGTCCCGGCCGGGCCGCGGGGAGCCTGGGTACTTGGAAGTCGGCTACGTGGACGACACGCAGTTCGTGCGGTTCGACAGCGACACCCCGAGTTCGGTGATGGAGCCGCGGTTGCCGTGGGTGGACCAGGAGGGGCCGGAGTAGTGGGAGCAGCAGACGCAGAACTTCAGGACCCGCACACAAACTCTTCAAGTGAGGCTGAATGAATTGCGTGTCCACTACAGCCAGAGCAAGGCCGGTGAGCGACCTGGGCCCGGGTCCAGGTCACGACCCCCGTCCCTACGGACGCGCCAAGGTCGCCCCGAGTGTCTGGGTCCTAGTGCCACGCTGAGTCTGCGCCCCACCCCCGTCCTGCGAATAGGTAAGAGCCCCTGGGGACTGTACTCGGTTTGGTTTTCAGTTTAGGCTTTAATGATTGCAGGTCAGGGCGGGGCCAGTGTCTCACACCTTCCAAAGGATGATAGGCTGCGACGTGGGGCCTGATGGGCACTGCCTCCGCCGTTACCTTCGTGAGGCCCACGACCGCGCGGATTACATCACCCTGAACCAGGACCTACGCTCCTGGACCATGGCGGGACACCGCGCCGCACATCACCCGCTGCAAGTGGGAGGCAGCCGGTGAGGCGGAGCGCTACAGGTACTACCTGGAGGGCACGTGCATGGAGTGGTTCCTCAAGTATCTGGAGATGGGG

>QURD01000174.1_Acinonyx_jubatus_68102-68787_exon2-3

CACACCGCGGTGTCCCGGCCGGGCCGCGGGGAGCCTGGGTACTTGGAAGTCGGCTACATGGACGACACGCAGTTCGTGCGGTTCGACAGCGACACCCCGAGTTCGGTGATGGAGCCGCGGTTGCCGTGGGTGGACCAGGAGGGGCCGGAGTAGTGGGAGCAGCAGACGCAGAACTTCAGGACCCGCACACAAACTCTTCAAGTGAGGCTGAATGAATTGCGTGTCCACTACAGCCAGAGCAAGGCCGGTGAGCGACCTGGGCCTGGGTCCAGGTCACGACCCCCGTCCCTACGGACGCGCCAAGGTCGCCCCGAGTGTCTGGGTCCTAGTGCCACGCTGAGTCTGCGCCCCACCCCCGTCCTGCGAATAGGTAAGAGCCCCCGGGGACTGTACTCGGTTTGGTTTTCAGTTTAGGCTTTAATGATTGCAGGTCAGGGCGGGGCCAGTGTCTCACACCTTCCAAAGGATGATAGGCTGCGACGTGGGGCCTGATGGGCACTGCCTCCGCCGTTACCTTCGTGAGGCCCACGACCGCGCGGATTACATCACCCTGAACCAGGACCTGCGCTCCTGGACCATGGCACATCACCCGCTGCAAGTGGGAGGCAGCCGGTGAGGCGGAGCGCTACAGGTACTACCTGGAGGGCACGTGCATGGAGTGGTTCCTCAAGTATCTGGAGATGGGG

>UIRR01000365.1_Arctocephalus_gazella_148830-148127_exon2-3

CTCACTGTCACGTCCCGGCCCGGCCGCGGGGAGCCCCGGTACGTGGAAGTCGGCTACGTGGACGACACGCAGTTCGTGCGGTTCGACAGCGACTCTGTCAGTCTGAGGTACGAGCCGCGGGCGCCGTGGATCGAGCGGGAGGGGCCGGAGTACTGGGACCGGGAGACGCGGGGCTCCAGGAATTGCGCACAGACTCTCCGAGGGAAGCTGAACGAAGTGCGCGGCTACTACAACCAGAGCGAGGCAGGTGAGCGACGCGGGCAGGGGACAGATCACGATCCCCATCCCCACGGACGGGCCGGGGTCGCCCCAAGTCTCCGTGTCCGAGCACCACCCCGAGGCTGCGGGACAGTGCCGTACCCCGAACGGGGAAGAACCCTTGCGGAGTCTACTGGGTTTGGTTTTCAGTTTAGGCTTTAATGACGATAGGTCGTGGTGGGGCGGGGCCAGGGTCTCACACCATCCAGAGAATGTACGGCTGCGACGTGGGGCCTGATGGGCGCTTCCTGCGCGGGTACTTTCGTGAGGCCTACGACGGCGCGGATTACATCGCCCTGAACGAGGACCTGCGCTCCTGGACCGCGGCGGACACGACGGCGCAGATCACCCGCCGCAAGTGGAAGGCGGCCGGTGAGGCGGAGTGGTGGCGAAACTACCTGGAGGGCACCTGCGTGGAGTACCTGCGCAGGCACCTGGAGCACGGG
