# Supplementary material for: Evolution of major histocompatibility complex class I genes in the sable Martes zibellina (Carnivora, Mustelidae)
Source: Ecol Evol. 2020 Mar 11;10(7):3439–49. doi: 10.1002/ece3.6140 (PMC7141072; doi:10.1002/ece3.6140)
Supplement: Supplementary file 10 — Legends [file ECE3-10-3439-s010.docx]

**Supplementary figures legends**

**Figure S1 Detailed information of primers.** The pair of primers (Mazi-MHCIex2F: 5’-GCTCCCACTCCCTGAGGTATTWC-3’; Mazi-MHCIex3R: 5’-GCGCAGCAGCGWCTCCTT-3’), which recognize highly conserved region of the MHC class I genes, were designed based on alignments consisting of the sequences from the NCBI and the sequences obtained from the first pair of primers. The sequences from the NCBI included (*Ailuropoda melanoleuca* [accession number: EU162660.1, NM_001304845.1], *Canis lupus familiaris* [U55029.1, NM_001020810.1, NM_001014767.1], *Felis catus* (EU153401.1), *Homo sapiens* [MH325442.1, MH325441.1, LT632317.1, MK140634.1]). The pair of primers (Mazi-MHCIex2F and Mazi-MHCIex3R) excluded the locus (*Mazi-MHCI*PS01–PS04*).

**Figure S2** **Alignment of amino acid sequences of presumably functional sequences in** *Martes zibellina.* The complete amino acid sequence of *Mazi-MHCI*01* is shown. Codon positions in α1 domain and α2 domain are numbered at the top. Dots (.) indicate consensus with the residues of *Mazi-MHCI*01.* Plus (+) denote an N-linked glycosylation site. Antigen binding sites (ABSs) which defined according to Bjorkman et al. (1987) are marked with gray boxes. Octothorpes (#) denote residues on an α-helix that is pointing away from the ABSs, inferred to interact with T-cell receptors (TCRs). Exclamation marks (!) denote residues pointing up on an α-helix, inferred to interact with interact with peptides and/or TCRs. Equal signs (=) denote conserved sites which bind the peptide N- and C- termini (Bjorkman et al., 1987). Disulphide bond formed between two cysteine ressidues is shown with a line spanning the cysteine residues.

**Figure S3** **Nucleotide sequence alignment of MHC class I intron 2 alleles identified in** *Martes zibellina.* The complete nucleotide sequence of *Mazi-MHCI*01* is shown; dots (.) denote identity with nucleotide positions in *Mazi-MHCI*01*; dashes (-) denote missing nucleotides. The degenerate 13-bp sequence motif (CCNCCNTNNCCNC) that is essential in crossover events at human recombination hotspots (Myers et al., 2008) is marked with gray boxes. Superscript numbers on alleles names indicate the number of MHC class I alleles in which a particular intron occurred: *Mazi-MHCI*01* (*Mazi-MHCI*04*); *Mazi-MHCI*02* (*Mazi-MHCI*03*); *Mazi-MHCI*05* (*Mazi-MHCI*06, Mazi-MHCI*07*); *Mazi-MHCI*09* (*Mazi-MHCI*10, Mazi-MHCI*12*); *Mazi-MHCI*PS01* (*Mazi-MHCI*PS03, Mazi-MHCI*PS04*); *Mazi-MHCI*PS06* (*Mazi-MHCI*PS07*, *Mazi-MHCI*PS08*, *Mazi-MHCI*PS09*, *Mazi-MHCI*PS10*, *Mazi-MHCI*PS12*).

**Figure S4** Maximum likelihood phylogenetic tree of MHC class I exon 2/α1 domain sequences from *Martes zibellina* and other carnivores. The support values greater than 75% were shown with circles. The sizes of the circles indicate the support values.

**Figure S5** Maximum likelihood phylogenetic tree of MHC class I exon 3/α2 domain sequences from *Martes zibellina* and other carnivores. The support values greater than 75% were shown with circles. The sizes of the circles indicate the support values.
